# Editorial: Plant nutrition and biostimulants: regulators of secondary metabolites and crop productivity in both normal and abiotic stress conditions

**DOI:** 10.3389/fpls.2024.1465149

**Published:** 2024-08-09

**Authors:** Probir Kumar Pal, Jana Šic Žlabur, Hong Wu

**Affiliations:** ^1^ Council of Scientific & Industrial Research - Institute of Himalayan Bioresource Technology, Palampur, India; ^2^ Division of Agricultural Engineering, Faculty of Agriculture, University of Zagreb, Zagreb, Croatia; ^3^ College of Life Sciences, South China Agricultural University, Guangzhou, Guangdong, China

**Keywords:** abiotic stress, biostimulant, crop productivity, plant nutrient, secondary metabilites

To meet the growing demands on food production and the production of biologically active phytochemicals, especially in terms of resilience to climate change, there is an urgent need to protect the environment and human health through a complete reversal of agricultural practices. Some plant groups, especially medicinal and aromatic plants (MAPs), are known to produce various bioactive secondary metabolites that have phytopharmaceutical properties. The main challenge for plant scientists is, therefore, to develop agricultural technologies that enable food and nutrition security and improve the production of secondary metabolites with the least possible negative impact on terrestrial ecosystems and human health.

In this perspective, plant nutrition and biostimulants play an important role in intensified agriculture. On the other hand, plant secondary metabolism involves complex synergisms and antagonisms, responses to environmental conditions, and relationships between the plant and the soil-water-atmosphere system, which are least understood. It is expected that unfavorable environmental conditions will radically affect plant productivity and plant secondary metabolism, but a methodological understanding of these effects is still lacking.

The judicious management of plant nutrients and the utilization of natural resources, particularly biostimulants, is a sustainable and environmentally friendly approach to alleviating abiotic stress by improving metabolic and photosynthetic processes. Therefore, within this Research Topic, efforts have been made to improve our understanding of the mechanisms related to the role of plant nutrients and biostimulants on plant productivity and plant secondary metabolism under both normal and abiotic stress conditions. Some important results from this research area are summarized here.

In this context, Zhang et al. reported the effects of different magnesium concentrations on the changes in leaf metabolites, photosynthetic fluorescence parameters, growth and quality of tea leaves. A total of 1240 metabolites were identified in the tea leaves; however, the total content of metabolites in the leaves was not significantly altered by applying Mg. Applying Mg at 0.4 mmol L^-1^ resulted in up-regulation of 654 metabolites and down-regulation of 586 metabolites compared to the control. It was also observed that the photosynthetic fluorescence parameters, theanine, and soluble sugar of tea leaves were increased with the higher Mg concentration, while the higher Mg concentration promoted the accumulation of polyphenols, caffeine and flavone. In addition, Mg promoted the growth of tea trees by improving the accumulation and metabolism of carbohydrate compounds. With this study, the authors have provided scientific evidence for the regulation of tea plant growth and quality by Mg.

García-García et al. evaluated the influence of chitosan oligosaccharides (COS) on artemisinin accumulation in *Artemisia annua* plants under drought. The applied concentrations of COS were 0, 50,100 and 200 mg L^-1^. There was no significant effect of COS on improving the growth of *A. annua* compared to the control under well-watered conditions. Dry biomass was also not improved by treatment with COS under drought stress, and the lowest value was observed when COS was applied at 100 mg L^-1^. The accumulation of saturated fatty acids (SFA), polyunsaturated fatty acids (PUFA) and unsaturated fatty acids (UFA) was altered by the application of COS under both well-watered and drought conditions. COS application reduced antioxidant enzymes, particularly APX and GR, as well as phenols and flavonoids under drought stress. However, the artemisinin content was increased by 34.40% by COS at a concentration of 200 mg L^-1^ compared to the control under drought stress. Thus, the present results indicate that artemisinin yield can be increased by applying COS under drought stress.

Humic acid (HA) is a proven biostimulant that increases the tolerance of plants to salt stress. However, the underlying mechanisms are not fully understood. Using advanced analytical techniques, Meng et al. elucidated the physiological and biochemical mechanisms of the effect of HA to increase salt tolerance in perennial ryegrass (*Lolium perenne* L.). The application of HA altered the important physiological traits (photosynthetic rate, transpiration rate, stomatal conductance, malondialdehyde, and Pro and intercellular CO_2_ concentrations), which are indicators of salt tolerance. The activity of antioxidant enzymes such as ascorbate peroxidase, catalase, peroxidase, and superoxide dismutase was reduced in ryegrass by applying HA. In addition, HA decreased the relative expression of the P5CS gene and its downstream products. This research provides important insights into the physiological, biochemical and molecular mechanisms of salt stress tolerance of ryegrass by applying HA.

Using innovative fertigation systems and improved nutrient composition, Velechovský et al. successfully improved the biomass yield and cannabinoid content of medicinal cannabis (*Cannabis sativa* L.). The recirculation system proved to be suitable in terms of total THC yield (sum of tetrahydrocannabinolic acid, Δ^9^-tetrahydrocannabinol, and Δ^8^-tetrahydrocannabinol). In this study, the appropriate time for harvesting has also been optimized.

The effects of plant nutrition on the nutrient profiles of stinging nettle (*Urtica dioica* L.) are not well understood. Therefore, Opačić et al. conducted an experiment to investigate the role of different nutrient solutions with different concentrations of macro- and micronutrients (especially N, P, K, Ca, Mg, and Fe) on the yield, nutrient profile and accumulation of specialized metabolites of stinging nettle at different harvesting stages in hydroponic systems. The interaction between the nutrient solutions and the harvesting stages on yield was significant, and the higher nutrient concentration resulted in maximum yield at the first cut. On the other hand, the nutrient solution with moderate concentration improved ascorbic acid, total phenolic, total non-flavonoid and total flavonoid content, as well as antioxidant capacity at the second cut. In contrast, a low concentration led to a higher accumulation of caffeoylmaleic acid, ellagic acid, ferulic acid, naringin and rutin trihydrate. Thus, they found that the hydroponic method is a possible alternative for the cultivation of nettle with high nutritional quality. In addition, the concentration of macro- and micronutrients in the hydroponic system was optimized for nettle cultivation.

In summary, this Research Topic provides insights into hitherto poorly explained mechanisms related to the role of plant nutrition and biostimulants in the production of plant secondary metabolites production under normal and abiotic stress conditions.

